# A Study of the Relationship Between Cystatin C and Metabolic Bone Disease in Preterm Infants

**DOI:** 10.4274/jcrpe.2088

**Published:** 2018-05-18

**Authors:** Sabriye Korkut, Şeyma Memur, Hülya Halis, Osman Baştuğ, Levent Korkmaz, Ahmet Özdemir, Tamer Güneş, Mehmet Adnan Öztürk, Selim Kurtoğlu

**Affiliations:** 1 Erciyes University Faculty of Medicine, Department of Neonatology, Kayseri, Turkey

**Keywords:** Cystatin C, metabolic bone disease, osteopenia, premature, speed of sound, renal failure

## Abstract

**Objective:**

Cystatin C (CysC) is commonly used as a marker of renal failure in premature infants. The aim of this study was to investigate serum CysC levels in osteopenia of prematurity (OP) and determine whether CysC could be safely used as a marker of renal insufficiency in infants with OP.

**Methods:**

Subjects were 50 preterm infants (≤32 gestational weeks). Calcium (Ca), phosphorus (P) and alkaline phosphatase (ALP) serum levels were measured in postnatal week nine, and bone density was measured concurrently by quantitative ultrasonography. Patients with a Z score of <-2 were considered to have OP.

**Results:**

The mean serum CysC levels in preterm infants in postnatal week nine were 1.50±0.19 mg/L. Serum CysC levels were not correlated with speed of sound values, Z scores, serum Ca, P or ALP levels. Serum CysC levels were not significantly different between infants with OP [1.50 (1.35-1.61) mg/L] and in infants without OP [1.58 (1.28-1.70) mg/L].

**Conclusion:**

The presence of OP does not affect the safety of CysC as a marker of renal insufficiency in preterm infants.

## What is already known on this topic?

Cystatin C is a valuable marker in the diagnosis of acute kidney injury in preterm infants. It was demonstrated that various parameters and conditions, such as respiratory distress, bilateral kidney anomalies, peripartum asphyxia, hemoglobin levels and sepsis, affect cystatin C values.

## What this study adds?

To our knowledge, this is the first investigation of the measurement of cystatin C in infants with osteopenia of prematurity compared to infants without osteopenia. The presence or absence of osteopenia of prematurity had no effect on measured cystatin C levels in our cohort.

## Introduction

Premature infants are at risk of developing bone disease due to a low bone mineral content. Neonatal rickets or osteopenia of prematurity (OP), also known as metabolic bone disease in premature infants, is one of the most frequent problems encountered in neonatal units. OP adversely affects linear growth and height in the long term, while causing fractures, growth retardation and respiratory problems in the short term (1). 

Dual photon X-ray absorptiometry (DEXA) is the gold standard method for the radiological measurement of bone mineral density. Nevertheless, time spent screening, artifacts caused by movement, exposure to radiation and cost limit the use of DEXA in newborns. A quantitative bedside ultrasonography (USG) assessment is a simple, inexpensive and noninvasive method, which can be used to obtain measurements related to bone mineral density and structure. Bedside assessment devices have been designed that quantify broad-band ultrasound attenuation or the speed of sound (SOS). Studies showed that quantitative ultrasound measurements correlated significantly with DEXA findings in both adults and children (2,3) and that they may be useful in evaluating structural and mechanical properties of bone (4). In preterm infants, a number of studies have demonstrated the potential clinical value of bedside ultrasound assessments of bone status (5,6,7).

Cystatin C (CysC) belongs to the cystatin family of cysteine proteinase inhibitors. It has a low molecular weight and is produced in virtually all nucleated cells in the body. The production rate of CysC does not change in inflammatory conditions (8,9) and it is commonly measured to determine the glomerular filtration rate (10,11,12).

Osteoclastic bone resorption depends on the activity of various proteolytic enzymes, particularly proteinases. CysC inhibits cysteine proteinase, a proteolytic lysosomal enzyme that prevents bone resorption. The association of CysC with bone metabolism has been demonstrated in a variety of *in vitro* studies (11,12). Clinical studies on bone metabolism and CysC are limited to adults, with no studies conducted in infants. Elevated serum CysC levels in postmenopausal women have been linked to increased bone fractures and reported to be a potentially promising biomarker for the risk of hip fractures (13,14).

Clinical studies of CysC in preterm infants have focused mainly on the relationship between CysC and renal function and aimed to determine the reference range. It was demonstrated that CysC is a valuable marker in the diagnosis of acute kidney injury in preterm infants (15,16,17,18). Other studies have demonstrated that various conditions, such as respiratory distress (19), bilateral kidney anomalies (20), peripartum asphyxia (21,22), abnormal hemoglobin levels (21,22), and sepsis (23), affect CysC values. 

Premature infants have an increased risk of both kidney failure and osteopenia, and the CysC level can be used as a diagnostic marker of renal failure in preterms. However, no studies have examined whether CysC levels are altered, and therefore reliable, for renal function assessment in premature infants with OP. Based on the literature, we hypothesized that the CysC level would be altered in OP. To shed light on this issue, this study investigated the relationship between CysC concentrations, bone density and levels of biochemical markers of bone metabolism [serum calcium (Ca), phosphorus (P), and alkaline phosphatase (ALP)] to determine whether serum CysC levels were altered in OP.

## Methods

Infants born between 24 and 32 gestational weeks who were admitted to the Newborn Unit of Erciyes University Faculty of Medicine were enrolled in the study. Infants with a severe congenital anomaly, congenital metabolic disease, perinatal asphyxia, who were diagnosed with acute renal injury or hypothyroidism up to postnatal age 9 weeks were not included in the study. Gestational week was determined by the last menstrual period of the mother. Infants with a birth weight below the 10^th^ percentile according to the Fenton 2013 chart were accepted to be small for gestational age (SGA). 

Clinical findings of OP become manifest between postnatal six and 12 weeks (1). In our subjects, bone density was assessed in the ninth week using quantitative USG. Serum Ca, P, ALP, creatinine and CysC levels were concomitantly measured. 

Serum CysC level was analysed on an Abbott Architect C 16000 (Abbott, US) analyzer and an enhanced nephelometric immunoassay. An automatic biochemical analyzer (Cobas 8000 c701, Roche, Mannheim, Germany) was used to determine the values of P (phophomolybdate), Ca (o-cresolphthaleine), and ALP (kinetic p-nitrophenilphosphate).

SOS was measured using a Sunlight Omnisense 2000 (Sunlight Medical, Tel Aviv) quantitative ultrasound sonometer. Measurements were done on the right tibia. The SOS measurement is based on the fact that ultrasound waves propagate faster in bone than in soft tissue. SOS is influenced not only by bone mineralization but also by quantitative factors, such as micro-architecture, elasticity and cortical thickness. The results are reported as meter/second. The SOS measurements are compared with mean SOS measurements of the same age group using a reference database, the Z score is automatically calculated based on the difference between the patients’ SOS scores and mean SOS scores of an age- and sex-matched group and expressed as a standard deviation by this sonometer. In this study, infants with Z scores <-2 were considered to have OP (7).

This study was approved by the ethical committee of Erciyes University Medical Faculty (02.10.2015/437). Informed consent from the parents of each newborn was provided.

### Statistical Analysis

Visual (histograms and probability plots) and analytical methods (Shapiro-Wilk’s test) were used to determine whether the data were normally distributed. Parametric data were presented as mean ± standard deviation. For intergroup comparisons, independent two-sample tests and the Mann-Whitney U test were conducted. Nonparametric data are presented as median values (25^th^ percentile to 75^th^ percentile). The correlation of serum Ca, P and ALP levels, in addition to SOS and Z scores, with CysC levels was determined by Pearson’s correlation analysis. The correlation analysis of the nonparametric data was tested by Spearman’s correlation analysis. A *chi*-*square test* was conducted to determine the relationship between categorical variables. In all the tests, the level of statistical significance was accepted as p<0.05.

## Results

In total 50 premature infants were included in the study. The demographic features of the infants and their mothers are summarized in Table 1. Only 11 of the 50 patients were still in hospital at post-natal age nine weeks for follow-up. Thirty-nine patients were evaluated during follow-up visits at postnatal age nine weeks.

The mean serum CysC level of the whole cohort of preterm infants was 1.50±0.19 mg/L. The median (25^th^ percentile-75^th^ percentile) and minimum-maximum serum creatinine levels of the whole cohort of preterm infants were 0.22 (0.19-0.29) mg/dL and (0.06-0.51) mg/dL, respectively.

Serum Ca, P, ALP, and CysC levels, in addition to SOS measurements, were grouped and compared according to gestational week and birth weight. In the group of infants with gestational ages of 26-29 weeks, the serum Ca levels (p=0.02), p levels (p=0.01) and SOS measurements (p=0.01) were significantly higher. There was no difference in the serum CysC levels of the infant group with gestational ages 26**-**29 weeks versus those of the infant group with gestational ages 30-32 weeks. In the comparison of infants according to birth weight, serum Ca levels (p=0.04) and P levels (p=0.02) were significantly higher, whereas serum ALP levels (p=0.04) were lower in those with birth weights ≥1500 g as compared with infants whose birth weight was <1500 g. There was no between-group difference in serum CysC levels according to birth weight (Table 2). The mean serum CysC level of boys and girls was 1.48±0.17 mg/L and 1.50±0.24 mg/L, respectively, with no significant between-group difference.

**Figure 1:**
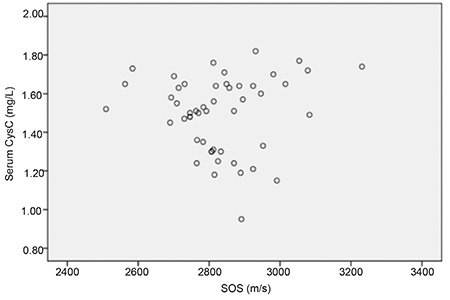
The serum CysC levels and SOS measurements of all infants CysC: cystatin C, SOS: speed of sound. There was no correlation between serum cystatin C levels and speed of sound measurements (Rho=0.04, p=0.75)

Serum CysC levels were not correlated with serum Ca, P and ALP levels or with SOS measurements (Figure 1) and SOS Z scores. Serum CysC levels were also not correlated with birth weight or gestational age. 

The SOS Z score values of 24 of 50 (48%) infants were <-2, and these infants were diagnosed with OP. Serum CysC levels were 1.50 (1.35-1.61) mg/L and 1.58 (1.28-1.70) mg/L in infants with OP and without OP, respectively, with no statistically significant different (p=0.34). The demographic and clinical features of infants with OP and without OP are summarized in Table 3.

## Discussion

The serum CysC level was not correlated with serum Ca, P and ALP levels or SOS measurements and Z score values. There was no difference in the serum CysC levels of infants with and without OP. Mean serum CysC levels in preterm infants in postnatal week nine were 1.50±0.19 mg/L.

Most previous studies in the English literature reported reference values for CysC levels in preterms in the postnatal first month (24). To our knowledge, there are no reports of CysC levels in preterm infants at nine weeks post-partum. Thus, this study aimed to provide reference data for CysC levels of preterm infants (born at ≤32 gestational weeks) in their ninth postnatal week.

Although some previous studies reported that serum CysC levels showed no gender difference in different age groups (17,18,25,26), others reported that they were higher in older males as compared with age-matched females (27). In the present study, although the mean CysC level was slightly lower in boys, we found no significant differences in serum CysC levels by gender. 

Previous research showed that body weight did not affect the serum CysC level (26,28). The findings of the present study were consistent with those in the literature, with no difference found in serum CysC levels between infants with birth weights above and below 1500 g. Serum CysC levels show a gradual decrease with term in preterm neonates. The levels are higher in preterm than term neonates, with the highest values found in the most immature cases (24). In the present study, at the postnatal ninth week, mean CysC values of infants of gestational ages 26-29 weeks tended to be higher than those of infants of gestational ages 30-32 weeks. However, this difference was not statistically significant.

In the current study, SOS and SOS Z scores determined by USG were lower in osteopenic infants. Previous studies reported that bone SOS measurements were lower in preterm infants than term infants during early postnatal life, with SOS values of preterm infants decreasing until age 2 months and not reaching the levels of term newborns until age 12 months when measured longitudinally (29,30,31). Previous research also reported that SOS values showed a significant association with birth weight and gestational age (29,30,32). In the present study, bone SOS values were lower in the infant group with gestational ages 26-29 weeks (p=0.01) and birth weights <1500 g (p=0.01). 

An SOS Z score of less than -2 suggests low bone density (7,33). In the present study, infants with a tibia Z score of less than -2 were considered as having OP and the demographic and clinical features of infants with and without OP were compared. According to previous estimates, OP occurs in 30-50% of infants with birth weights <1000 g and in 23-32% of infants with birth weights <1500 g (34,35). In the present study, 24 of the 50 (48%) patients were diagnosed with OP. The high rate of OP in our study may be explained by the timing of the SOS measurements, which were performed when the infants were two months old, a time when SOS values are lowest in premature infants. Previous studies reported that the incidence of OP was inversely correlated with gestational age and birth weight (34,35). In the present study, SOS values of infants were reduced in those with lower gestational weeks and lower birth weights. When the patient population was evaluated according to the presence or absence of OP, the gestational age and birth weight of infants with OP were lower than those without OP, but there was no statistical significance. This may be due to the Z score values, which were similar in both groups, as well as the small sample size.

Previous *in vitro* studies confirmed that CysC prevented bone resorption (36,37,38). In one study, Lerner and Grubb (36) showed that CysC in bone culture stimulated with parathyroid hormone and parathyroid hormone-related peptide was a potent inhibitor of mineral mobilization and matrix degradation. In an osteoblast cell culture system, Danjo et al (37) demonstrated that CysC affected bone morphogenetic protein signal pathways in osteoblasts, causing variation in osteoblasts and enabling mineralization and bone formation. Stralberg et al (38) showed that CysC inhibited osteoclast differentiation and formation. Clinical studies of bone resorption of CysC are limited to adults. Elevated serum CysC levels reported in postmenopausal women have been linked to an increased risk of bone fractures (39,40). Based on the results of these previous studies, we speculate that changes in the CysC level may act as a protective mechanism in OP and play a role in the pathogenesis of bone resorption. 

In the present study, we investigated the relationship of serum Cys C levels with bone density and levels of biochemical markers of bone metabolism (Ca, P, and ALP). The results showed that serum CysC levels were not correlated with serum Ca, P and ALP levels or with SOS measurements and SOS Z score values.

### Study Limitations

Assessment of osteopenia with QUS alone is a limitation of the current study. In subsequent studies, CysC levels should be investigated in patients with bone status assessed with DEXA.

## Conclusion

CysC levels are not altered in OP. The presence of OP does not affect the safety of CysC as a marker of renal insufficiency in preterm infants.

## Figures and Tables

**Table 1:**
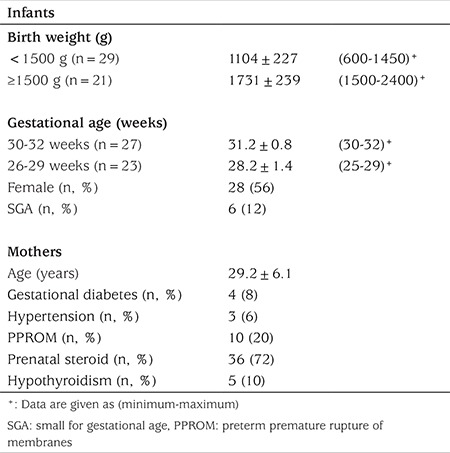
The demographic features of the infants and their mothers

**Table 2:**
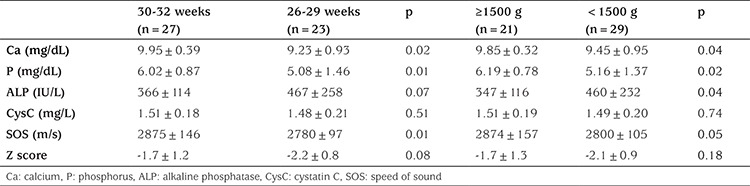
Measurements according to grouping by gestational week and birth weight

**Table 3:**
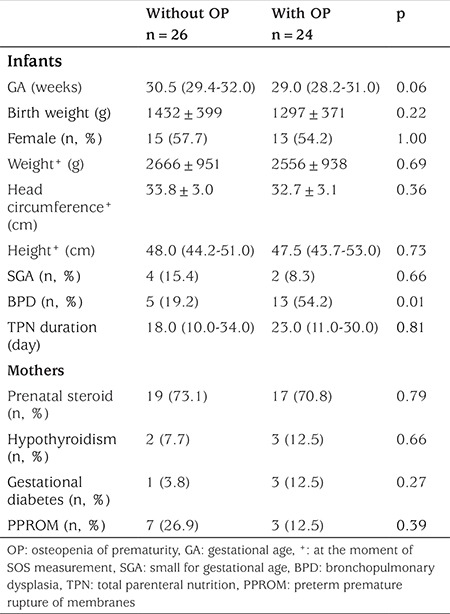
Demographic and clinical features of infants with osteopenia of prematurity and without osteopenia of prematurity
